# Can resistance testing improve outcomes for children and adolescents with HIV?

**DOI:** 10.1016/S2214-109X(24)00262-6

**Published:** 2024-08

**Authors:** Julie H Levison, Catherine Orrell

**Affiliations:** Division of General Internal Medicine, Division of Infectious Diseases, Department of Medicine, and Mongan Institute, Massachusetts General Hospital, Boston, MA 02114, USA; Harvard Medical School, Boston, MA, USA; Desmond Tutu HIV Centre, Institute of Infectious Diseases and Molecular Medicine, and Department of Medicine, University of Cape Town, Cape Town, South Africa

The global scale-up of HIV antiretroviral therapy (ART) has generated substantial individual and public health benefits. Viral suppression restores immune function and halts HIV transmission. Progress for children and adolescents has been more delayed than it has been for adults. As of 2022, 1·5 million children aged 1–14 years were living with HIV.^1^ Global estimates of viral suppression vary substantially by region, but show lower rates of viral suppression in children compared with adults. In a large cohort analysis of people who initiated ART between 2010 and 2019, adjusting for missing viral load data, 59% of those younger than 18 years had viral suppression at 3 years after the initiation of ART compared with 65% of adults.^2^

HIV drug resistance can diminish ART efficacy and the probability of viral suppression. Causes of resistance in children and adolescents include suboptimal ART adherence, food and drug interactions, and improper weight-based dosing. In low-income and middle-income countries, additional factors include exposure to non-nucleoside reductase inhibitors as part of the prevention of mother-to-child HIV transmission, ART supply shortages, insufficient laboratory and clinical capacity to receive and integrate viral-load and drug-resistance results into individual care, and climactic disasters, political conflict, and emerging infectious diseases outbreaks that can destabilise access to health services.^3^ Globally, the clinical effect of genotype resistance testing (GRT) for children and adolescents with HIV is unknown because public health programmes widely use dolutegravir, an integrase strand transfer inhibitor with a high genetic barrier to resistance.^4^

In *The Lancet Global Health*, Jennifer Anne Brown and colleagues present the Genotype-Informed Versus Empirical Management Of Viremia (GIVE-MOVE) trial,^5^ examining whether children and adolescents with HIV and viraemia benefit from the addition of GRT versus usual care, consisting of adherence counselling, viral-load monitoring, and empirical clinical decision making. The trial was conducted in Lesotho and Tanzania, two sub-Saharan countries that are hyperendemic HIV areas with modest public-health resources. Lesotho and Tanzania have excelled at reaching the UNAIDS HIV prevention and treatment goals by 2025, but lag in viral suppression rates in children and adolescents.^6^ The GIVE-MOVE trial can contribute to a larger global conversation on the role of laboratory-informed HIV clinical care and the combination of biomedical, sociobehavioural, and structural interventions needed to realise the health benefits of ART for children and adolescents with HIV.

The trial analysed 284 participants (144 in the GRT group and 140 in the usual care group), aged 6 months to 19 years, across ten clinical centres and had low rates of loss to follow-up, with five (3%) participants in the GRT group and four (3%) in the usual care group. The design, implemented in real-world settings, allowed for standardised window periods to assess outcomes. The primary outcome was death, hospitalisation, a new WHO HIV clinical stage 4 event, or the absence of a viral load of less than 50 copies per mL within the 36-week study period. The authors found no significant difference between GRT and usual care (67 [47%] participants in the GRT group *vs* 73 [52%] in the usual care group; adjusted odds ratio 0·79 [95% CI 0·49 to 1·27]; adjusted risk difference −0·06 [95% CI −0·17 to 0·06]; p=0·34).

GRT did not alter clinical and virological outcomes for this cohort, of which 274 (96%) had WHO stage 1 disease. Given that lower CD4 counts are associated with emergence of drug resistance,^7^ the effect of GRT might have been more pronounced in those with late-stage disease and could be a key area of focus for country programmes.

The study excluded 26 (18%) participants in the GRT group and ten (7%) in the usual care group from the primary analysis due to a delayed or absent treatment-decision visit. Qualitative research with participants not engaged in the study but able to be tracked in follow-up could offer valuable feedback on intervention development. Existing evidence-based interventions for the engagement of youth in HIV care, such as integration of peer workers and novel digital adherence applications, provide a scaffold from which to incorporate GRT without deterring youth.^8–10^

The GIVE-MOVE trial underscores the complexity of ART adherence in this population: of the 84 participants in the GRT group with successful GRT, 32 (38%) had no major drug-resistance mutations at viraemia, implying that recent ART non-adherence was the most likely driver of HIV viraemia. Almost half (129 [45%]) of the participants had at least one deceased parent. Adherence interventions that address adverse childhood experiences and integrate the family unit and social support will assure that children and adolescents receive the services they need as programmes move to simplify and tailor services, such as in the implementation of long-acting ART.^11^

All participants were to receive enhanced adherence counselling over three visits during 9 months, but the number and duration of adherence visits that participants actually completed (ie, dose of behavioural intervention) or the extent to which the delivery of the adherence content matched the expected protocol (ie, fidelity) is not clear. Data on adherence dose and fidelity would help explain the relationship of the adherence intervention effect on the outcomes.

The clinical benefit of GRT at viraemia in sub-Saharan Africa, a region with two-thirds of the global HIV prevalence, remains uncertain. The GIVE-MOVE trial presents a road map for future effectiveness and implementation trials to identify the population of children and adolescents with HIV to receive GRT and the types of interventions needed at the individual, clinic, and health-system level.
